# A Practice-Inspired Mindset for Researching the Psychophysiological and Medical Health Effects of Recreational Dance (Dance Sport)

**DOI:** 10.3389/fpsyg.2020.588948

**Published:** 2021-02-25

**Authors:** Julia F. Christensen, Meghedi Vartanian, Luisa Sancho-Escanero, Shahrzad Khorsandi, S. H. N. Yazdi, Fahimeh Farahi, Khatereh Borhani, Antoni Gomila

**Affiliations:** ^1^Department for Language and Literature, Max Planck Institute for Empirical Aesthetics, Frankfurt am Main, Germany; ^2^Department of Psychology, University of Tehran, Tehran, Iran; ^3^Dresden Frankfurt Dance Company, Frankfurt am Main, Germany; ^4^Shahrzad Dance Company, San Francisco, CA, United States; ^5^3Fish Corporate Filmmaking, Istanbul, Turkey; ^6^Institute for Cognitive and Brain Sciences, Shahid Beheshti University, Tehran, Iran; ^7^Department of Psychology, University of the Balearic Islands, Palma, Spain

**Keywords:** wellbeing, brain, emotion, mindfulness, aesthetic emotion, recreational dance, hobby dance

## Abstract

“Dance” has been associated with many psychophysiological and medical health effects. However, varying definitions of what constitute “dance” have led to a rather heterogenous body of evidence about such potential effects, leaving the picture piecemeal at best. It remains unclear what exact parameters may be driving positive effects. We believe that this heterogeneity of evidence is partly due to a lack of a clear definition of dance for such empirical purposes. A differentiation is needed between (a) the effects on the individual when the activity of “dancing” is enjoyed *as a dancer* within **different dance domains** (e.g., *professional/”high-art”* type of dance, *erotic* dance, *religious* dance, *club* dancing, *Dance Movement Therapy* (DMT), and what is commonly known as *hobby, recreational* or *social* dance), and (b) the effects on the individual within these different domains, as a dancer of the **different dance styles** (solo dance, partnering dance, group dance; and all the different styles within these). Another separate category of dance engagement is, not as a dancer, but as a spectator of all of the above. “*Watching dance*” as part of an audience has its own set of psychophysiological and neurocognitive effects on the individual, and depends on the context where dance is witnessed. With the help of dance professionals, we first outline some different dance domains and dance styles, and outline aspects that differentiate them, and that may, therefore, cause differential empirical findings when compared regardless (e.g., amount of interpersonal contact, physical exertion, context, cognitive demand, type of movements, complexity of technique and ratio of choreography/improvisation). Then, we outline commonalities between all dance styles. We identify six basic components that are part of any dance practice, as part of a continuum, and review and discuss available research for each of them concerning the possible health and wellbeing effects of each of these components, and how they may relate to the psychophysiological and health effects that are reported for “dancing”: (1) rhythm and music, (2) sociality, (3) technique and fitness, (4) connection and connectedness (self-intimation), (5) flow and mindfulness, (6) aesthetic emotions and imagination. Future research efforts might take into account the important differences between types of dance activities, as well as the six components, for a more targeted assessment of how “dancing” affects the human body.

## Introduction

*“Wherever the dancer steps, a fountain of life will spring from the dust.”—*Rumi

In the past 20 years or so, empirical research in psychology and affective neuroscience has started to report important psychophysiological and medical health effects for individuals who practice “dance.” Especially, longitudinal assessments suggest that “dance” outperforms other types of recreational activities in terms of their health enhancing potential, including ball sports, crosswords, swimming, etc. (King et al., 2003; Verghese et al., 2003; Merom et al., 2016). However, in meta-analytic assessments and reviews about intervention studies that specifically use “dance” to target different mental and physical health-related variables yield an inconclusive picture, piecemeal at best (Bradt et al., 2015; Mansfield et al., 2018; Karkou et al., 2019; Millman et al., 2021). We believe that varying definitions of what constitutes “dance” have led to this heterogenous body of evidence.

As a recreational practice (or sport), dance is gaining momentum, slowly reaching the attention of policy makers and stakeholders in health disciplines. However, a more practice-informed approach to dance would help this endeavor greatly.

Different dance styles may have differential effects on the practitioner, depending on each style’s specific features. Heterogeneity in movement form, practice features, music and movement intention of different dance types should be taken into account. As for any empirical research, we must identify the components (or parameters) of a phenomenon, and then research them systematically. As put by Hauser et al. (2007), “*parameters can be added or subtracted in order to determine which parameters contribute most significantly to the output*” “output” in the case of dance being the important health and wellbeing effects that have already been observed for some dance practices.

To give an example, comparing the effects on individuals of practicing say, *Haka* dance (an energetic group fight dance), with *Ballet* dance (a highly athletic and technically very complex dance style, that often tells a story, mostly with no touch between dancers, but with important demands of synchronicity), with *Greek Sirtaki* (a group dance with generally simple movement patterns, danced at social events for shared enjoyment, cardio as an exercise, performed usually holding hands, or shoulders between dancers), or with *Salsa* dance practice (a partnering dance with constant touch and proximity of one single partner) is impossible without a clear consideration of the variables that differentiate these activities, even if they are all called “dance.”

These dances share some basic important components which we will also review in section “Dance *Domains* and Dance *Styles—*and Psychophysiological Effects” of this paper, because they surely are beneficial in themselves for human psychological and physical health. The very different movement patterns of these dance styles, exertion levels, emotional tone, amount and type of social contact, cognitive demand, movement intention and music type yield a whole different set of challenges, opportunities and demands for the dancer that are relevant for empirical research. There are also similarities between dance styles, and it is often a question of degree. Therefore, we also review important similarities between the dances of the world, to motivate a more targeted approach also to these similarities (section “A Wheel of Dance”).

With the help of dance professionals, we first outline a differentiation between (a) the effects on the individual when the activity of “dancing” is enjoyed *as a dancer* within **different dance domains** (*professional-“high-art”* type of dance, *erotic* dance, *religious* dance, *club* dancing, *Dance Movement Therapy* (DMT), and what is commonly known as *hobby, recreational* or *social* dance), and (b) the effects on the individual within these different domains, as a dancer of the **different dance styles** (solo dance, partnering dance, group dance; and all the variants within these). Another separate category of dance engagement is, not as a dancer, but as a spectator of all of the above. “*Watching dance*” has its own set of psychophysiological and neurocognitive effects on the individual (section “Dance *Domains* and Dance *Styles—*and Psychophysiological Effects”), that depend on, e.g., the context where dance is witnessed.

Next, we outline similarities between dance styles, and identify six basic components that are part of any dance practice, to a larger or lesser degree, as part of a continuum. We review and discuss available research for each of them concerning the possible health and wellbeing effects of each of these components, and how they may relate to the overall health effects that have been reported so far for “dancing.” These are (1) **rhythm and music**, (2) **sociality**, (3) **technique and fitness**, (4) **connection and connectedness** (self-intimation), (5) **flow and mindfulness**, (6) **aesthetic emotions and imagination** (section “A Wheel of Dance”).

## Dance *Domains* and Dance *Styles*—and Psychophysiological Effects

It is firmly established that dance is an artform. However, in addition to “being an artform,” dancing puts special physical demands on the body, like no other artform. Therefore, the dance practitioner is both an artist and an athlete (Koutedakis and Jamurtas, 2004). To see dance as only and artform and not a sport (and vice versa) is purist and considering today’s scientific evidence, somewhat ignorant. This multi-layered nature of dance is, in essence, what makes dance a unique type of activity, even if domains and styles within the broad umbrella term of “dance” may vary. This special combination of creative artistry and physicality may make “dance” an important utility for health promotion for individuals (Millman et al., 2021). Please note that we do not consider any of the now following attempts at definition as orthogonal or otherwise exclusive categories, but as situated on continua of various parameters that together constitute what we may label as “dance.”

Therefore, a first important differentiation is in order. Dance can be practiced within very different **domains** and with different intentions. Intentions and the motives that inspire a person to dance vary greatly between recreational dance, and other dance domains.

For instance, in *professional dance*, the intention is to perform for an audience and express specific states (or even to create different unspecific states in the audience just with the transmission of abstract choreographic information, not always linked to a clear narration), emotions and sometimes stories through dance movements. In *erotic dance*, the emphasis is on movements that seduce and entice. In *religious dance*, the intention is to connect with a divine force or agent. In *club dancing*, the intentions of individuals to go dancing can vary greatly (get out of the house, socialize, find a mate). *Dance Movement Therapy* (DMT) is a therapy form, taking place in well-controlled clinical settings for purposes of therapy and personal growth. Another, separate domain of dance, is the perspective of the audience, of those *watching* dance, researched by Reception Theories in Performing Arts, and by empirical and neuroaesthetics in the empirical sciences.

All of these domains happen in very varied locations and social settings, that all have their own socio-cognitive variables that need to be part of the assessment of any psychophysiological effects of “dancing,” since these more domain-specific variables are likely to contribute in their own way to the effects. Dance doesn’t happen in a vacuum, and certainly the borders between these domains can be fuzzy, depending on the preferences of the dancer. However, for empirical research, these domains must be considered and delineated, depending on the objective of any experiment.

### Dance Domains

Like other professional domains (e.g., in other sports domains), **professional dance** practice puts additional pressures and demands on the dancer’s mental and physical health, and will not be considered within this piece (see e.g., Koutedakis and Jamurtas, 2004; Shah, 2008; Twitchett et al., 2009; Nordin-Bates et al., 2011; Mainwaring and Finney, 2017, for different demands on the professional dancer, including challenges relating to physical injury, eating disorders and body dissatisfaction, etc.). One important aspect that differentiates recreational vs. competition/professional dance sport is that performative dance is generally evaluated in terms of *how it looks*, while recreational dance is evaluated as much as possible in terms of *how it feels*, a fact which may explain why recreational and professional movement practice have differential effects on practitioners’ emotion regulation (Gruzelier et al., 2014; Zajenkowski et al., 2015). This also has repercussions on hormonal signatures and the psychological appraisal mechanisms of dancers (Rohleder et al., 2007; Quiroga-Murcia et al., 2009; Quiroga-Murcia et al., 2010; Quested et al., 2011; Berndt et al., 2012). Very little work is available about this, although it is very important to understand more about how to achieve the emotion and immune regulatory effects of dancing (and what aspects of a dance practice might impede them—e.g., variables related to performance and competition).

Likewise, ***erotic* dancing** will not be considered within this paper, as it has its own psychological, physiological and social complexities (Maticka-Tyndale et al., 2000; Footer et al., 2018). Unfortunately, comparisons of human dance with dance-like behaviors in the animal kingdom have led to some confusion. The latter occur in the animal kingdom exclusively in courtship contexts for mate attraction. However, such comparisons have led to misunderstandings regarding human dance, as being an activity for sexual seduction only (Christensen et al., 2017b). An example of such misconceptions about some dance styles is the “Cabaret Dancer” that started to conquer the stages world-wide in the early twentieth century. Too often has the public eye confused this type of dancing with *erotic* dances for attraction and seduction with considerable socio-economical costs for dancers (Shay, 1999; Mefthai, 2017). This is not to say that dance cannot have an erotic component. As we mention above, the boundaries between the different domains are fuzzy, and may need to be delineated clearly when in an empirical context.

It is very likely that dance has had important ritual and religious functions since the dawn of human civilizations. **Religious or sacred dance** is its own special domain where dance is used as a special way of connecting with a divine force or agent (Vinesett et al., 2015; Peoples et al., 2016), often related to religious or ritual experiences (Kaeppler, 1981; Hanna, 1995, 2010; Pušnik, 2010; Fischer et al., 2013). Cognitively, but also emotionally, such dance experiences are qualitatively, if not also quantitatively different from recreational dances.

**Club dancing** is also to be considered a category of dance by itself. It is mostly entirely improvised and does not follow any specific technique or personal development path. Club dancing is an important activity for emotional catharsis and time out for many on a Saturday night. However, it also comprises unique psychosocial elements related to the club scene and the particular environment of night club dancing (Kurtz et al., 2011; Schifferstein et al., 2011; Gripenberg-Abdon et al., 2012; Ellamil et al., 2016; Beselia et al., 2019), that may at times obstruct health benefits related to dancing in other domains (Foley et al., 2019). This domain of dance requires separate assessment and will not be considered here.

“**Dance Movement Therapy**” (DMT) is a dance “style” that has been developed by psychologists, medical doctors, and dancers as an integral type of dance training that should help people in the clinical domain to gain health on various psychological and physiological health effects (Koch et al., 2014; Millman et al., 2021). However, also here, the evidence spanning from interventions with DMT is very heterogeneous, potentially due to the very different types of clinical problems it is being applied to, ranging from depression to cancer to behavioral problems (Jeong et al., 2005; Duberg et al., 2013; Bradt et al., 2015; Ho et al., 2016, 2018; Karkou et al., 2019; Koch et al., 2019; Predovan et al., 2019). We will not include DMT in the present article.

**Watching dance** is another category of dance engagement that merits a separate “domain,” even if, of course, “watching dance” is profoundly intertwined with any dance activity. Dance spectators all over the world join dance performances to *watch* dance, without dancing themselves, or, dancing alongside. Population-scale survey data about health and arts engagement have illustrated that there may be health and well-being benefits for individuals from merely attending arts events (as opposed to participating in them), including attending museums, concerts, cinemas, and also “watching dance.” While these studies currently group “watching dance” in with other arts activities (Fujiwara et al., 2014; Wheatley and Bickerton, 2017), future research may address the question about the effects of watching dance regularly, in particular, on individuals’ health and well-being.

From the perspective of neuroscience, “watching dance” has become a hot topic since seminal studies in the mid-2000 used dance movements as stimuli to uncover important neurocognitive evidence about how movement expertise shapes the human Action Observation Network (Calvo-Merino et al., 2005a, 2006; Cross et al., 2006, 2009). Research in this domain is concerned with how the human brain processes complex actions (e.g., dance moves; Vaessen et al., 2018) in laypeople and in experts (Calvo-Merino et al., 2005a, b; Cross et al., 2006; Jola et al., 2012, 2013; Jola and Grosbras, 2013; Kirsch et al., 2015, 2018). The aesthetic, affective, and social components of a “dance watching” experience are multilevel and have also attracted much attention. Especially, from the perspective of empirical and neuroaesthetics, an endeavor preoccupied with understanding the processes that are at play when human observers consume and engage with art objects, activities, situations and materials that can give rise to aesthetic appreciation (Chatterjee, 2003, 2011). Empirical and neuroaesthetics of dance is a relatively recent endeavor, as compared to other arts domains (visual arts, music, film, etc.; Christensen and Calvo-Merino, 2013; Calvo-Merino and Christensen, 2014; Christensen and Jola, 2015; Kirsch et al., 2016). One important question for this discipline with regards to dance movements is whether and how dance viewers understand emotions and intentions of a dance movement (Jola et al., 2011; Grosbras et al., 2012; Kirsch et al., 2013; Christensen et al., 2014, 2016a,b, 2018; Bachrach et al., 2016), what audiences find aesthetically pleasing in a dance movement (Calvo-Merino et al., 2008, 2010; Cross et al., 2009; Daprati et al., 2009; Miura et al., 2010; Orgs et al., 2016), how audience members’ bodies synchronize with each other (Pollick et al., 2018), or with the dancer during the performance (Bachrach et al., 2015; Theodorou et al., 2019). Interindividual difference measures that illustrate different reasons why people choose to dance, and which dance styles are preferred are also recently gaining momentum (Maraz et al., 2015a, b), as is research into dance style preferences for watching dance by people with different personality profiles (Jola et al., 2014). Cognitive, affective and social effects of regular “watching” of dance have received little empirical attention so far (though see Jang and Pollick, 2011). Likewise, interindividual difference measures about who dances what and why is also still rather elusive. However, these are clearly areas ripe for expansion (Sevdalis and Keller, 2011).

**FIGURE 1:**
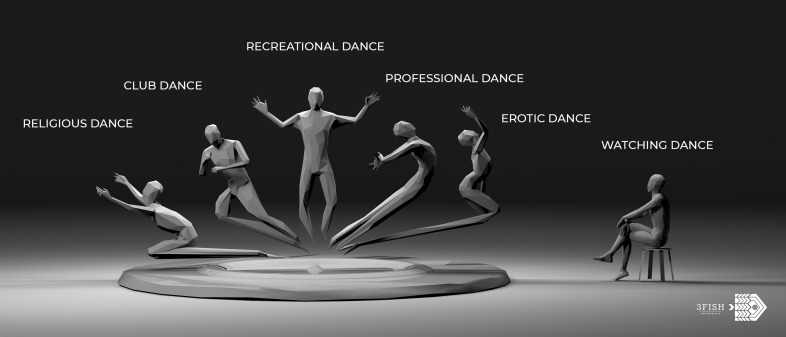
Domains of dance practice and enjoyment. Professional dance, club dance, religious, and erotic dance, and Dance Movement Therapy (DMT) all also contain the 6 elements. However, they also possess additional variables that can be more complex to analyze in terms of their health effects, often related to the level of physical exertion required (and risk for injury), and/or the social context that can be hostile or otherwise complex (competitiveness, drugs, predatory, aggressive). Of course, it is possible that these different domains of dancing overlap or that they are part of a continuum (e.g., a professional dancer of ballet may go club dancing and their dance is then infused by ballet technique, however, here used in a completely different domain), depending on the choices of each dancer. Many dances can be sexualised, depending on the intention of the dancer and of the viewer (e.g., in belly dance that can be both a dance used for personal growth, but also is often misunderstood as a form of attraction). Some people perceive dance in a club as a religious experience, and within any of these domains one can chose to become a professional dancer (except maybe for club dancing). However, these domains should each be considered and analyzed each in their own right, depending on where the dance happens, their usage, intention, movement patterns, and psychosocial variables distinguish them. © Sina HN Yazdi, 3fish.co.

See Figure 1 for an illustration of the different dance domains.

The illustration of the domains in this figure may be seen as a hierarchy or a classification, with recreational dance as a domain of dance somewhat elevated, because it can include all of the other domains. The proximity to the watcher of the different dance domains can be seen as a measure of the importance that an audience may have in a given domain of dance for the effects that it may produce. The latter two points are the expression of the imagination of the creator of this figure. © Sina HN Yazdi; artist/filmmaker, 3fish.co.

In addition to the above differentiations of dance domains, let us turn to the differentiations that are required within the recreational dance domain.

### Dance Styles—*Same, but Different*

Dance styles of the world share some components that we will review in section “A Wheel of Dance.” However, they vary greatly on other aspects, and so may any psychophysiological effects that may result for individuals. This fact is not being sufficiently taken into account by the empirical sciences. Dances may differ in terms of:

(i) **Interpersonal contact during the dance, and type of contact**. The level of interpersonal contact varies between individual/solo dances, partnering dances, group dances, and of dances that encompasses in a mix of all the above. For example, the recreational training regime of classical ballet or Persian dance includes half to two-thirds of the class dedicated to practicing technique and expressivity individually (or, “solo”). Then follow sequences as group dances, couple or triplet dances, where synchronicity is very important. Dancers don’t necessarily touch during these dance episodes. Folk and traditional dances are usually performed in a group, or couple dances with hand, finger or shoulder holding. Partnering dancers mostly train with their partner in a duo, and occasionally “solo,” but mostly with uninterrupted touch between dancers.

As we will review in section “Sociality, Culture and History,” touch has special effects on the human body. Therefore, dance styles that involve much touch, and dance styles that do not, are not necessarily comparable at a psychophysiological level. Besides, some dances involve practitioners to synchronize their movements between each other, while others do not. Given the important effects of synchrony on the human brain, the type of interaction is an important variable to take into account, as we discuss in section “Sociality, Culture and History.”

(ii) **Physical exertion**. The level of physical exertion and effort required varies greatly between dance styles (Koutedakis and Jamurtas, 2004; Rodrigues-Krause et al., 2015). Ballet, Haka dance, capoeira, step dance, Zumba, etc. are high exertion dances and may have different effects on the human body than dances that do not rely on high complexity and cardio workout features, see section “Technique and Physical Fitness Effects.”
(iii) **Cognitive demand.** The cognitive demand of a dance style is related to the level of complexity of the movements to be learned. Dances like Ballet and Persian solo dance have a very complex movement repertoire that needs to be learned, like the vocabulary of a language. Some other dance styles are much less complex in terms of the vocabulary of movements to be learned. See section “Technique and Physical Fitness Effects.”

**FIGURE 2:**
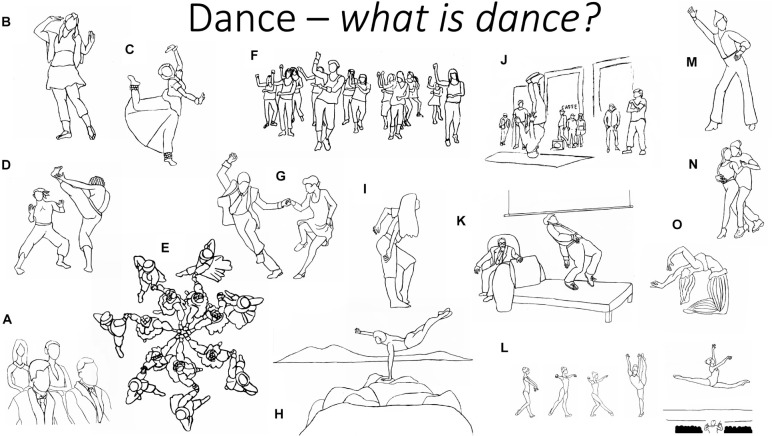
Illustration of non-exhaustive list of dance styles and usages. Dance can be **(A)** something you watch as a spectator, **(B,C)** an artform and sport that connects you with your culture (e.g., Persian, Indian dance), **(D)** a fight dance (e.g., Capoeira, Haka), **(E)** something you do as a group (e.g., folkdances, other group and traditional dances), **(F)** a type of fitness activity (e.g., Zumba), **(G)** something you do with a partner (e.g., Argentine Tango, Swing dance, Ball room and Latin dances, Salsa, Bachata, Kizomba, etc.), **(H)** something you do on your own, as self-intimation, a fitness enhancer, to come to terms with emotions or situations, mood management, **(I)** as a form of free expression of your feelings, **(J)** an activity that connects you with a specific sub-culture (e.g., Hip hop, lyrical, Cabaret, tap dance), etc., **(K)** a therapy with a dance movement therapist, **(L)** a performative art form that you do in front of an audience, **(M)** something to do free style in a club dancing context, **(N)** something you do for intimacy and seduction in a couple dance, **(O)** as an *erotic* dance forms (e.g., some types of pole dancing, Belly dancing—please note that these can be practiced both for professional purposes and for purposes of self-intimation with no seductive or erotic intentions of the dancer). © JF Christensen.

**Ratio choreography/improvisation.** Dance styles vary in terms of how much choreography/improvisation they include. A dance can be fully improvised i.e., it relies on no pre-choreographed steps or technique (e.g., club dancing). The other extreme would be a dance that is only performed following a fixed choreography (e.g., the Ballet Nutcracker is danced exactly the same, with the same steps, all over the world). Recreational dances differ in terms of how much improvisation is commonly engaged in by practitioners, using pre-choreographed steps/technique/vocabulary of a dance style. For instance, Argentine Tango only has about 5 basic steps that are combined repeatedly over and again, according to the improvisational desire of the dancers. Persian solo dance or classical Ballet repertoires have hundreds of movement-units or basic steps that are combined improvisationally too. However, these dance styles have a much larger level of constraints regarding the combinations than Argentine tango, making them more demanding cognitively. Folk or traditional dances are group dances and mostly rely on simple patterns, usually following one leader that indicates the next sequence of movement, although there is a large variation in terms of movement and improvisational complexity (compare, e.g. some Central Asian group dances, with American Square dance and with *Rueda Cubana* [a very complex Cuban social dance]). All of the latter are social group dances that rely on the direction of one leader that calls out the next movement sequence. Different dance styles—within the recreational dance domain—may therefore have differential physical, emotional and cognitive effects on practitioners, and even within a dance style these can vary. See Figure 2.

All the named points in this section differentiate dance practices. But they can also be found simultaneously in others, for instance, in a classical and/or contemporary choreographed ballet. All of this is a matter of degree or continuum. There are no exact categories. However, the dances of the world vary along these variables, and empirical evidence from neurobiological research shows that these variables (Interpersonal contact, Physical exertion, Cognitive demand, and Ratio choreography/improvisation) have differential effects on the human body and cognitive systems when controlled systematically in other domains than dance. Thus, they must also be taken into account in empirical research with “dance.” Therefore, for empirical research, it is important to collaborate with dance practitioners that are sensitive to the specific features of dance practice that scientists might have to consider for their experimental design involving “dance.”

Future research may want to work toward a “differential diagnostics” between dance forms, regarding their specific psychophysiological effects. “Differential diagnostics” between styles is unfortunately not yet possible, since comparative studies are very scarce. In what follows we will give a brief overview of the heterogeneity of evidence.

### A “Differential Diagnostics”?

We hope that the proposed practice-based framework may trigger targeted research into dance sport as a recreational and health-promoting activity, *informed by dance practitioners*. Longitudinal studies have shown that dance sport as a hobby is an important protector against diseases that are very costly from a personal and socio-economical point of view, including heart disease and dementia (Verghese et al., 2003; Merom et al., 2016).

Several positive effects of hobby dance sessions have been reported in the empirical literature of psychology and affective neuroscience. However, no clear pattern is emerging. Sometimes “dance” research uncovers important health effects, and sometimes it shows nothing at all. This is likely due to some heterogeneity in the dance styles used, and due to the way participants are assigned to experimental groups.

In the naturalistic context, individuals may self-select into specific dance practices that bring them desired effects (e.g., on dependent variables including health effects, mood management, etc.). However, another person with other personal characteristics may not derive the same effects from that same dance practice. Personal preferences for a dance style choice may play an important role too. Person A may find ballet right for them, while another prefers swing dance. Much research in experimental psychology has shown that personal preferences modulate important variables that determine the success of any intervention, including motivation and compliance. This is a topic to consider when we assign participants to intervention groups “randomly.”

Besides, lack of conclusive results can also be due to a poor selection of control groups for intervention-type studies using “dance.” As mentioned above, different dance styles put different physical, cognitive and emotional demands on the dancer (even if they happen on a continuum rather than in categorical terms), and control groups must be identified that are as closely aligned to these characteristics, while not being a dance (e.g., other movement practices including sports, movement meditations, etc.). This is particularly important if researchers have specific hypotheses about the contributions of individual parameters of a dance style to specific enhancements (dependent variables). For example, some dance styles include backward walking while others don’t. As we will see later, regular backward walking may lead to some cognitive benefits. Empirical work would now need to establish whether the parameter “backward walking” in a dance style makes the practice of that particular dance style outperform an activity chosen as control condition that includes backward walking.

We will give three examples for such divergent findings that need follow-up research with valid control conditions to allow for a “differential diagnostics” in the future: research shows that (i) some dance styles seem to increase salivary cortisol, others lead to the opposite, (ii) some dance practices seem to increase bone density, others lead to the opposite, and (iii) some dance styles seem to lead to a better attentional focus, and other cognitive enhancement, however, other studies are unable to find such cognitive effects.

Recreational dance episodes have been found to decrease cortisol in the blood, while professional, competitive dance episodes increase the stress hormone (Rohleder et al., 2007; Quiroga-Murcia et al., 2009, 2010; Quested et al., 2011; Berndt et al., 2012). However, the true picture seems more nuanced than that. It is well-known that the idea “high cortisol = bad vs. low cortisol = good” is too simplistic, and using different types of dance and dance domains may help to shed light on this question. One study compared two types of recreational dance practice: Haka dance and Japanese dance. After the sessions, differential activation patterns were observed (both subjective and objective) between the two (Kuroda et al., 2017). After Haka dancing, participants had higher levels of cortisol and felt more energized than after a session of the Japanese dance, after which participants felt poised and calm, and their salivary cortisol levels had decreased. A similar differential pattern was observed in a comparative study between an African group dance and another movement practice, Hatha Yoga (West et al., 2004). After the African dance session, participants felt energized and their salivary cortisol had increased, as compared to the Hatha Yoga session, where it decreased and participants reported completely different affective effects. The hormone cortisol is often related to stress responses, however, comparative studies with different dance styles may help to clarify any beneficial effects that a heightened activation pattern may have. Participants of all groups in these studies with recreational dance reported important emotion regulation effects after the sessions, suggesting outright positive effects of any of the dance practices, despite the differential salivary cortisol profiles. Targeted assessments of which dances produce what type of psychobiological effects would be beneficial for making the right choice for empirical assessments of psychophysiological and neuroendocrine effects of “dance.”

A similarly inconclusive pattern emerges if we compare studies that assess whether or not “dancing” is related to increases or decreases in bone density. While some studies with Waltz dance show increased bone density (Young et al., 2007; and see also Kudlacek et al., 1997; Matthews et al., 2006), a study assessing dancers of ballet dancers’ found the opposite (Amorim et al., 2015). An assessment of what is causing these differences would be helpful, taking into account different domains and styles of dance, and what behavioral patterns within the dance culture might trigger these effects, other than the movement practice itself (e.g., eating patterns, social comparison processes, etc.), instead of speaking about “dance” in general.

A similar “differential diagnostics” on dance styles might be useful in terms of what is causing cognitive enhancement effects that are found after some dance intervention but not others. Some studies have reported increases in concentration and attention in participants of dance interventions, yet, again, results are mixed (Cofini et al., 2018; Levin, 2018; van den Berg et al., 2019). Thus, it may be the case that some dance styles have features that help dancers develop their attentional capabilities, while other dance styles train other cognitive abilities, but not attention. For example, one movement aspect (of many!) to focus on would be to analyze whether the dance style includes backward walking. Studies with children and adults with ADHD seem to suggest that episodes of backward walking develop patients’ attentional capabilities (Viggiano et al., 2015). Comparing the effects of dance interventions with dance styles that include substantial proportions of backward walking vs. dance styles that do not would be helpful (e.g., compare Argentine tango that includes substantial amounts of backward walking for the follower, to Bachata dancing which mainly involves sideward walking for both partners). Besides, comparative efforts should be made to assess whether the complexity of movement patterns (e.g., Persian solo dance [very complex] vs. Siritaki [not complex]), impacts post-intervention attentional abilities.

### Clinical Considerations

The heterogeneity of the field of “dance” research is also leading to very heterogeneous results of dance interventions in the clinical domain, where dance has been applied as therapy with very limited success only (Levine and Land, 2016; Millman et al., 2021). However, if we lack a clear understanding of what causes desirable emotional, cognitive and physiological health effects *through different dance practices*, applying any dance style to any clinical domain feels somewhat blindfolded, and could be optimized, once we understand the mechanisms better.

For example, some research points toward the differential effects of dance styles to improve movement-related problems. One comparative study showed symptom reduction for participants with Parkinson’s Disease after an Argentine Tango course, while a Waltz course did not yield such effects (Hackney and Earhart, 2009, 2010). As discussed by the authors of the latter study, one reason for these differential results might be in the specific movement patterns of the dance styles. Argentine tango involves many stops and movement re-initiations (which is particularly difficult for people with Parkinson, so practicing it, aided by the rhythmic cues of the music, might be why Argentine Tango is helpful). Another point is that Argentine Tango involves movement cues for the body that calls the body to action, e.g., a foot that stops the other foot, and such cues are particularly helpful for patients with Parkinson to help them initiate gait. Besides, in Argentine Tango the follower walks backward most of the time, and backward walking has been shown to improve balance in general (Hao and Chen, 2011), which is another issue that Parkinson patients struggle with. Waltz on the other hand, does not have these features to the same degree, it moves continuously with no designated stops and pauses. On the other hand, other comparative studies have shown that Waltz dancing can have positive results for people with other clinical conditions. One study compared a Waltz course intervention to a traditional exercise intervention in the recovery phase of patients in a hospital after a stroke. The group that underwent the Waltz course improved their cardiovascular symptoms over and above the traditional exercise and control group (Belardinelli et al., 2008). But what is it about the different partnering dances (Argentine Tango, Waltz, Ballroom, etc.) that have good effects, and for which movement disorder? And which dances are good for other types of clinical problem, such as heart disease?

The authors of this article, too, believe that dance can help individuals recover from clinical levels of dysfunction. However, different clinical populations would likely benefit from different dance practices. Thus, also, using hobby dances (e.g., Argentine tango, ballet, ballroom, hip hop, etc.) as a therapy for clinical conditions (cancer, trauma, Parkinson, dementia, ADHD, etc.), still needs substantial empirical backup from research in healthy populations to be able to formulate clear hypotheses about why the choice of one specific dance style (and not another) might be beneficial for a specific health problem. Such targeted assessments are currently not common practice, and researchers seem to choose “dance” as one specific thing, which it is not. This heterogeneity makes the results appear hopelessly inconclusive and piecemeal, certainly deterring policymakers from targeted investments into the health effects of dance practice as a recreational activity with important psychophysiological and health effects.

## A Wheel of Dance

Dance is perhaps the most multi-dimensional of all art forms. Visual arts such as painting and sculpture happen in space, whereas music happens in time. Movement happens in both space and time simultaneously. The dancer physically senses their own emotions, the motion in her/his body, often in connection with an external sound or an internal drive to dance (a feeling, a sensation, a thought, etc.), and in addition, dance often happens in connection with sensing the body of one or several other dancers around (Mettler, 1947; Kostelanetz and Anderson, 1998). To dance is a multisensory, multicomponent experience.

However, there are also universal features of dance, all over the world (Hejmadi et al., 2000; Jola et al., 2012; Christensen and Calvo-Merino, 2013; Jola and Grosbras, 2013; Christensen et al., 2016b). Usually, dance (i) happens to a rhythm (often music), (ii) involves learning a technique (series of steps and movements, a movement vocabulary of a style), which stimulates mental and physical fitness, (iii) sociality (social interaction with other dancers and a broader dance community, its culture and history), (iv) a sense of connectedness (with oneself, a partner, with the community, the music, and/or with a greater whole, e.g., “nature”), (v) requires mindfulness (mental focus and concentration on the here and now) and triggers flow (a state of consciousness that is characterized by loss of time and space perception, mental relaxation and enjoyment), and (vi) produces different types of imagery (imagination, fantasy), and aesthetic emotions that can range from a simple change of current mood state, to life changing emotional insights (happiness, sadness, attraction, awe, being moved, etc.).

Dance practitioners and teachers might be interested in information about the scientific evidence that is currently available about different types of dance, and in particular, about the six components that all dances share. Dancers have intuitively used these six in their practice ever since, without any need for science. However, mutual cross-fertilization between arts and sciences can lead to progress in both domains. We hope that pointing out the specific benefits that practitioners can obtain by aiming their dance practice at obtaining these 6 components through their practice, this might encourage dance practitioners to share this knowledge within the communities of dancers; and also, importantly, to share these with new recreational dancers, to open doors into this exciting type of activity and hobby, that is dance.

The authors of this article became aware of a teaching resource that is being used by several dance teachers in the Argentine tango community, called the *Wheel of Dance* (Argentine tango teacher couple, Natalia and Agustin^1^, based in La Plata, Argentina, and Tango School Oh Lala in Luzern^2^, Switzerland). The basic idea is that the practice of any dance style should include, not only the learning of specific steps but careful consideration of 6–7 different components that the training should focus on equally. We set out to review the scientific literature about these different components from an interdisciplinary point of view. Our team includes psychologists, neuroscientists, and dance practitioners from several different dance styles (Persian dance, Ballet, Armenian folk dance, contemporary dance and Argentine tango). We review available scientific evidence about each of these different components for individual health and well-being when used from a recreational dance perspective, plus we include experience reports from two professional dancers from our team.

Dance practice of any style, be it Russian ballet, Persian dance, Spanish Flamenco, Armenian Kochari, Argentine Tango, Kurdish Halparke, or Cuban Salsa, involves six basic components: (1) **music and rhythm**, (2) **sociality**, (3) **technique and physical fitness,** (4) **connection and connectedness**, (5) **flow and mindfulness**, (6) **aesthetic emotions and imagination.**

These six components *unite* the dance styles of the world, although each dance style has its own specific sub-components which should be taken into account, especially for empirical research into the health and well-being effects of dancing, as referred to in the section “Introduction.” Each of the six components may be more or less strongly represented in a dance style and this may affect any psychophysiological and medical health effects that can be expected from an intervention with this style. We review available evidence of some psychophysiological and medical health effects of each of them in this section, and each section will be accompanied with a text box with experience reports from the professional dancers on our team.

**FIGURE 3:**
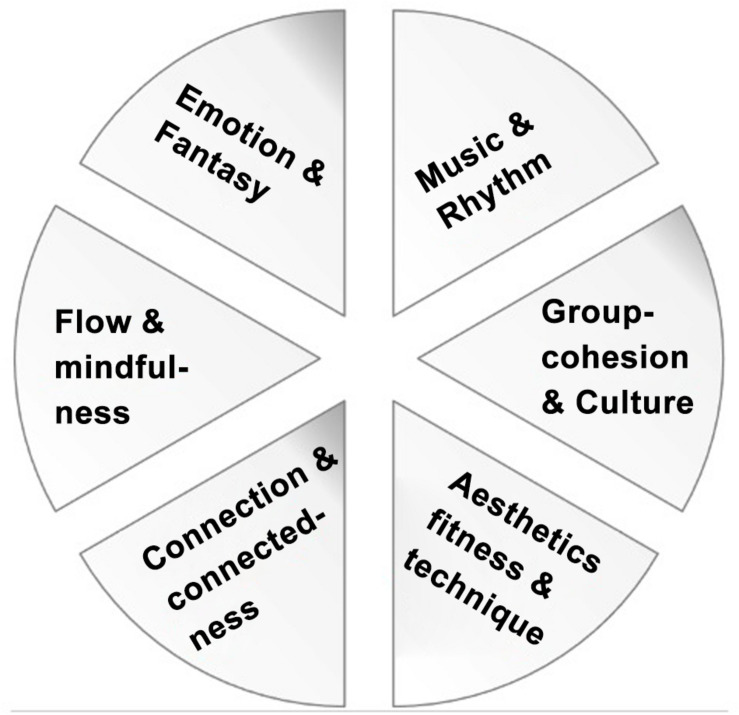
The components for any dance style are: (1) **rhythm of the music of this style**, (2) **sociality, culture and history behind it**, (3) **technique and fitness effects that it involves,** (4) **connection and connectedness experienced while dancing (self-intimation)**, (5) **flow and mindfulness during training**, (6) **imagery and aesthetic emotions triggered by the practice, and as part of it.** We propose the “Wheel of Dance” as a cognitive tool to illustrate these six components, both to empirical researchers, as well as for dance practitioners and teachers: If each part of the wheel receives equal amounts of attention in class, the wheel will be round and fulfill its function as a wheel: roll, and transport us to a new place, for our mental and physical health and well-being. Knowing about these 6 healthy components of dance practice might help practitioners aim their dance activities more specifically toward achieving these goals, and thus, enhance the efficiency of their practice, and the well-being within the practice, and beyond. For a more practice-based approach about how to teach this, see the web pages of this Argentine tango teacher couple, Natalia and Agustin, based in La Plata, Argentina, and of Tango School Oh Lala in Luzern, Switzerland. Dance teachers of other styles may take from this analysis the importance to teach students equally in all components, and how to avoid aspects of dance practice and the dance community that are detrimental for their (and their students’) mental and physical health. Thinking of these six intertwined components as the six parts in-between the spokes of a wheel offers an excellent metaphor for illustrating the different elements and how they should be considered in equal terms, and explains why each of them has its own relevance and importance, both for practice and for empirical research.

Please see Figure 3 for coarse illustration for the Wheel of Dance.

### Music and Rhythm

Recreational dancers are exposed to considerable amounts of music, and coordinate their movements and their emotions to the musical stimulus for hours on end.

Professional dance may happen in time and space alone, without the accompaniment of music. Especially in the Western contemporary dance scene, it is often proposed that dance is not necessarily dependent upon music (Stevens et al., 2009; Jola et al., 2014). However, in the hobby/recreational dance domain, dance is mostly associated with music, and in most traditional dance forms, and in many contemporary cultures, music and dance are practiced together, and the strong connection and harmony between them is a vital part of the practice (this is not to mean that a dancer from any domain, the recreational domain included, cannot suddenly start dancing, in response to an internal drive, feeling, sensation or thought, without the need for music at all; see Box 1).

**Box 1.**

Dance and music.

**View from a dancer: a dance without music?**

*Life is based on rhythm. We vibrate, our heart pumps blood. We are a machine with rhythm, that’s what we are* (Van der Kolk, 2014).

One very common aesthetic expectation that exists toward dance is the following: dance should be depending on music. Let’s be clear about this point, dance is a fully independent form of art, and it doesn’t need any other art form to exist. Think about it. *Dancers can be the engines of music* (paraphrasing William Forsythe). Our movements create our own rhythm and this gives a positive self-reassuring feeling for people who start experimenting with dance. While we dance, we create different possibilities of rhythm, speed and *tempi* (of course, normally following a choreographer’s indications but not always). My personal experience as a professional dancer in choreographies without music was always a very fulfilling one. I could focus internally in my coordination and mechanics in between/with my limbs and different parts of my body much more. It became always a kind of internal meditation, following my own rhythms – the ones I was creating on my doing.

*Luisa Sancho Escanero; Dresden-Frankfurt Dance Company, Germany*.

**View from a dancer: how to “get” the music of a new style?**

As in most cultures, Persians dance is common at social gatherings, and in this case, the dancing is always accompanied by Persian music. How the dancer hears and relates to the music is a big part of the cultural experience and emotional expression. When teaching Persian dance to non-Persians, I often have to analyze the music and explain dynamic cultural nuances that happen in the music and it is always very interesting for non-Persians to understand and experience these nuances. The most common Persian rhythm is in the 6/8-time signature, and it is accented and syncopated in a special way that makes it very recognizable as “Persian,” and also difficult for non-Persians to hear. Many Westerners cannot figure out when to snap their fingers or clap to the beat because they cannot quite find where the beat is, or they clap on the wrong part of the rhythm. The Persian 6/8 is accented on the first and the fourth beats, so that the rhythm creates a swing. However, the melody is so syncopated on top of the rhythm that it is difficult to hear the meter. Yet, once you do hear it the swing quality becomes very playful and irresistible to dance to.

*Shahrzad Khorsandi; Shahrzad Dance Company, San Francisco, United States*.

The human auditory system has evolved in such a way that our species is the only one capable of complex motor entrainment (e.g., tapping along to the rhythm of a beat), and of categorization of auditory input into “chunks.” One explanatory hypothesis about this is referred to as the “audio motor evolution hypothesis.” It implies that large scale projections via ganglia connect the auditory cortices of the human brain with the muscular systems, enabling complex rhythmical behaviour including speech and dance (Merchant and Honing, 2014; Ackermann et al., 2014; Honing and Merchant, 2014; Merchant et al., 2015). The motor and auditory systems of the human brain are narrowly interrelated and, in some instances, sounds are in fact translated into electrical signals that travel to the muscles and result in motor potentials (for important studies see: Paltsev and Elner, 1967; Xenos et al., 1976; but see also: Thaut et al., 1999, Thaut, 2003; Molinari et al., 2003; Zatorre et al., 2007; Schmahmann and Pandya, 2009; Kornysheva et al., 2010; Cesari et al., 2014; Mainka, 2015; Olshansky et al., 2015; Poikonen et al., 2016). Thence, likely the human drive to groove along any rhythm that presents itself in our environment (Bernardi et al., 2018).

Music itself has a very special effect on our body, including immunoregulatory effects (Fancourt et al., 2014; Sachs et al., 2015; Finn and Fancourt, 2018); and see for sad music: (Frey et al., 1981; Messmer, 2009; Sachs et al., 2015). Besides, interaction with other people through music has been shown to have several remarkable social effects, including enhancing emotional empathy in 8–11 years old children (Rabinowitch et al., 2013), and increasing spontaneous helping and cooperative behavior among children and adolescents (Kirschner and Tomasello, 2010), and adults (Kniffin et al., 2017; Ruth, 2017). Moreover, shared musical experiences increase group cohesion, affiliation, collective thinking, social inclusion (Welch et al., 2014), and cross-cultural understanding (Brown, 2000; Cross and Morley, 2008; Clarke et al., 2015). Furthermore, research shows that music listening can have positive effects on emotion regulation in infants (Trehub et al., 2015), and playing and listening to music helped university students improve their emotional awareness and regulation (Saarikallio and Erkkilä, 2007; Van Goethem and Sloboda, 2011; Dingle and Fay, 2017). Interestingly, research shows that only dance in conjunction with music, but not without music, lowers levels of cortisol in blood (Quiroga-Murcia et al., 2009), a finding that has been associated with subjective stress reduction and immunoregulatory effects.

For any dance practice that is danced to music, its music is an integral part of it. Rhythmical patterns vary greatly between dance styles and cultures. See for example, Box 1 for a description from Persian dance.

Future research: The neurocognitive and neurohormonal effects of music on health and wellbeing are being explored. What is lacking is a targeted, differential assessment of (1) how this exposure contributes to health and wellbeing effects in the dance domain (enhancement of healthy function and treatment of dysfunction) and (2) what effect different types of music and rhythmic patterns in the different dance domains may have.

### Sociality, Culture and History

Learning a dance style as a recreational activity comes with an increase in social contact. Dancers relate to other people during the dance class, in social dance evenings, in festivals, etc. This affords many opportunities to simulate others’ state of mind and others’ movements. Therefore, the ability to “simulate others” is “practiced” extensively in the dance world and should be considered an important experience-based component that differentiates people that dance from people that don’t.

Sociality (associating with others in groups), is a key driver of mental and biological health and well-being for individuals. The Polyvagal Theory by Steven Porges explains the careful interrelations between the social environment and inner medium of the human body. Friendly and safe social interaction in “our” group can help activate the body’s relaxation response, promoting cell reparation, immuno-regulation, and metabolic- and digestive regulation. This happens through the facial feedback loops via the cranial nerves that receive input from the motoric activity of our facial muscles in facial expressions that happen while we navigate a social situation (Porges, 1991, 1992, 1995, 2007, 2009; Porges et al., 1994; Carter et al., 2008). This happens, of course, regardless of which “group” is yours, but the point is that recreational dance affords a high quantity of high-quality opportunities for direct interactions with likeminded people.

Another mechanism by which beneficial effects of sociality come about is through an affective touch mechanism. Ct-cells are a special type of receptor in hairy skin (Olausson et al., 2016). These are a special neural interconnections with systems of the body responsible for homeostatic regulation and immunoregulation. Trustful social interactions with touches and hugs are therefore likely to have important health benefits for individuals (Heinrichs et al., 2003; Keysers et al., 2004; Hansson et al., 2009; McGlone and Spence, 2010; Morrison et al., 2011; McGlone et al., 2014; Lloyd et al., 2015). One study showed that having regular hugs reduced participants’ risk of developing a cold after controlled exposure to an innocuous respiratory virus (Cohen et al., 2015), and having a hug after an argument was related to more positive affect in subsequent days (Murphy et al., 2018).

Dancing, and the dance communities afford very varied opportunities of sociality, where dance communities can become more or less tightly knit social networks of mutual support and friendship (Maraz et al., 2015a; Lakes et al., 2016; Schwender et al., 2018). It is thought that dance has filled such social functions ever since humans started to live together in social groups (Radcliffe-Brown, 1922; Evans-Pritchard, 1928; Schögler and Trevarthen, 2007; Dissanayake, 2009). Historically, and in all cultures, dance has been a social activity, a form of stress relief, or a type of community ritual (Kaeppler, 1981; Hanna, 1995, 2010; Pušnik, 2010; Fischer et al., 2013). This continues to be the case in both rural and urban areas of contemporary society (Shay, 1999, 2002; Neveu-Kringelbach and Skinner, 2014; Gholami, 2016). See Box 2 for an example of support from dance communities.

**Box 2.**

Dance communities in Corona times.

**Dance communities in Corona times**

For an example of bonds within dance communities: During the Corona virus lock down 2020, there has been a massive online demand from the dance communities, for dance contact, presence and practice online. From professional companies offering their dancers online trainings, to dance schools transforming their program completely online to the creation of online platforms to guarantee different kind of daily trainings by professional dancers themselves. The opportunities for dance classes go beyond boarders, time zones and other limits.

For Swing dance check out Dax & Sarah’s Rhythm Juice and Jo & Kevin’s iLindy from California. For Argentine Tango, Argentine teacher Raquel Greenberg in London offers both couples’, leader and ladies’ technique online. For Persian dance and some Central Asian dance classes, go to Pomegranate Garden Dance. You can dance with the Batsheva dancers by tuning in to Gaga Online Classes with Ohan Naharin in TelAviv and New York City. For Ballet and more, check the online offer of the *Centre of Dance an der Kulturbrauerei* Oliver Detelich, Berlin, Germany. Or, go to *Dance Live Europe* in Basel, Switzerland with Jorge Garcia Perez. And don’t forget to check out the *Dancing Classrooms* with Paul Dulaine from the wonderful movie *Dancing in Jaffa.* For a new app, try the one by Tanzschule Gutman from Germany. There’s plenty out there now, just google it. And of course: don’t forget to support your local dance community by taking their online classes! You *can* dance in your living room.

Another mechanism by which dance facilitates psychological and physical health effects is imitation and simulation. Imitation is at the core of the human ability to learn such intricate movements as a pencil grip to pick up a pencil and write, as well as for a ballerina to go on pointe and spin the night away. This capacity for simulation and imitation gives us the ability to simulate other minds and movements too (Noy et al., 2011), and thus, to learn from them (Freedberg and Gallese, 2007; Shafir et al., 2015; Sumanapala et al., 2017). Dancers practice this mental and movement simulation of others repeatedly. In fact, in both recreational and professional dance domains, it is common practice to ask new students to place themselves behind the best students of the class and to copy. Over and again.

Therefore, on a daily basis, and in high quantity and quality, dance students imitate the movement of the teacher and others in the class, and their partner or group during dancing and they must understand the minds and movements of the others during the dance parties for a successful dance experience (milongas, socials, etc.). This ability of our brain to simulate and then to imitate using the body is why we’re able to learn from others, why we understand others’ mental states (Theory of Mind), and why we can feel what the other feels (empathy). Research investigating differential effects of empathy in dance and non-dance populations are still relatively heterogenous. However, measuring the amount of simulation (copying) that a person is engaging in, in terms of quantity and quality, may help shedding light on this question.

Several studies show that participating in synchronous movement engages action mirroring mechanisms in the human brain, the action observation network (Cross et al., 2006), which seems to be one possible basis for the phenomenon that individuals bond after a dance session (Schmidt and Richardson, 2008; Tarr et al., 2014, 2015). A group of people moving in unison feel more personally connected to each other afterward (Hove and Risen, 2009; Launay et al., 2014), conform more to each other’s opinions, and like each other more than groups that haven’t moved in unison together (Reddish et al., 2013; Codrons et al., 2014; Tarr et al., 2015; von Zimmermann et al., 2018), and develop a more prosocial behavior, for instance, evidenced by more efficient cooperation on a post-dance task (Wiltermuth and Heath, 2009; Valdesolo et al., 2010; Fischer et al., 2013; Lumsden et al., 2014). You can dance together for 3 min and you know each other much better than after 10 years of working together (Nazemi, 2020). Dancing together creates strong bonds between us.

Another mechanism by which dance has social bonding effects is through the facial mirroring of emotions, touches, and hugs that happen both during dancing, and within the dance community. The social context of dance classes and social dance evenings afford important opportunities for social exchange, both verbal and biochemical. Neurohormonal bonding mechanisms involving oxytocin and prolactin are implied in these bonding effects (Dunbar, 2010; Algoe et al., 2017).

Additionally, dance training helps to develop social skills because dance comes with a set of social etiquette rules and protocols that make this activity a type of multilevel social hobby (Novack, 1990; DeMers, 2013). Studies show that dance training encourages the development of group awareness, self-discipline, impulse-control and, respect for others (Lobo and Winsler, 2006; Staiano et al., 2018). For example, a study showed that a creative dance program could enhance the level of social competence among pre-schoolers, while also reducing their behavior problems (Lobo and Winsler, 2006). In another study, Staiano et al. (2018) showed that the experience of dance exergaming is linked to subjective health, stabilized peer conflicts, and pleasurable physical activity in adolescent girls (Staiano et al., 2018).

Hobby dance affords countless opportunities for social interaction, simulation, imitation, touch, complex emotional exchange and social reward. However, of course, negative experiences during the dance, including competitiveness, negative social comparison processes (e.g., jealousy), social exclusion and so on during dance practice or in the dance community more generally, are very relevant because they release stress-related biochemicals in the body, which may impede important health effects otherwise associated with dancing. These processes may vary, depending on the dance community. Future research could help to disentangle the various roles played by negative and positive affect in such protective potential of “dance.” Here, we reiterate the importance of distinguishing between professional dance practice and different hobby practices, and between other sports or meditative practices (see e.g., Ermutlu et al., 2015; Mansfield et al., 2018; Vrinceanu et al., 2019). See Box 3.

### Technique and Physical Fitness Effects

Recreational dancers practice cognitively demanding technical skills of full body movement coordination, in combination with emotion regulation and engage in different levels of physical exertion while doing so. Dance includes complex movement coordination of trunk, arms, legs and head, complex spatial navigation, memory and implementation of movement sequences stored in memory into new variants, and, importantly, all this happens in coordination with the music, and often the interaction and coordination with a partner, or group, requiring high levels of multisensory integration. Of course, humans use their full body every day, however, in dance training the body is twisted and bent in new ways, and steps are done in ever new combinations. This makes dance practice qualitatively and quantitatively different on the cognitive side from many other recreational practices. Besides, dance training regimes are much less repetitive, and require more new combinations than most other recreational activities. This is obviously a question of degree and not of absolute categorical terms, neither for dance, nor for other recreational activities.

All these might constitute the brain “work out” aspect of dance practice, which is related to important cognitive effects in hobby dancers and professional dancers alike (Bläsing et al., 2012; Kattenstroth et al., 2013; Rehfeld et al., 2018), though future assessments might focus specifically on what aspects of dance practice yield which cognitive effects (for mixed results, see Niemann et al., 2016), to target them more efficiently, also in the domains of cognitive problems, including Parkinson disease, dementia, ADHD, etc., as discussed in the introduction.

Every formal dance style has a technique, sometimes even several techniques developed by different teachers and choreographers (e.g., ballet: Russian Waganova style, Danish Bournonville style, Italian Cecchetti style, etc.). There are always particular dynamic and expressive qualities within the aesthetic parameters of distinct movement repertoires (Peterson Royce, 1978; Hanna, 1995, 2010; Hejmadi et al., 2000; DeFrantz, 2005; Anakesa and Jeannin, 2008; Christensen and Calvo-Merino, 2013; Ramaprasad, 2013; Christensen and Jola, 2015). The technique of a dance style is a vocabulary through which the dancer can then express him/herself through the specific language of that dance style, what s/he feels. For hobby dance, the effect of the dance on any audience becomes secondary. Especially for recreational dance, the dance can simply be a means of emotional expression, and self-intimation for the dancer (Orgs et al., 2013; Christensen et al., 2017b, see also component 6, section “Aesthetic emotions and the imagination.”).

The technique aspect of dance practice is one of the aspects that could be hypothesized to be at the core of the cognitive effects of recreational dancing. Contrary to what may be assumed, recreational dancers spend considerable time on learning the technique of their dance style. The technique can mean learning steps and placement of the body in space, but also involves mastering many subtle micro movements of the body, e.g., how to place the foot on the ground (e.g., heel-ball (tango) or ball-heel (ballet), learn to transpose pressure during step from inside heel to outside toes, etc.), how to turn in (or out) the hips during movement, how to use head movements for balance (e.g., spotting during pirouettes), etc.

Another important point here is the “Marking” experience (Warburton et al., 2013). “Marking” is something that dancers engage in on a daily basis. They go through dance movements, without dancing them full out, reducing and minimizing the physical effort almost to zero while going through the choreographic material with the body, focusing on the mental activity. Therefore, for example, a dancer can reproduce mentally a choreography lifting their legs and prolonging their hips and legs much more than in reality while they use the mental imagery (Karin, 2016; Karin et al., 2016). Their quality of movement is optimized by this process, it becomes very articulated, and they don’t tire their muscles as much as they would if they would dance out the movements full out each time.

Practicing such complex motor actions in connection with music and often a partner or group constitutes an important challenge for the brain, and an opportunity for neuroplasticity. One longitudinal study found that dancing was a protective factor against developing dementia in old age (Verghese et al., 2003), and dance has been shown to trigger the brain to form new interconnections (Hänggi et al., 2010; Karpati et al., 2016; Gujing et al., 2019), improve memory (Zhu et al., 2018), attention (Coubard et al., 2011), and the ability to multitask and to plan (Alpert, 2011; Hamacher et al., 2015). Interestingly, another cognitive effect that has been found is that, especially, when the dance form connects us with our own culture and the aesthetics that it comes with, it is intrinsically motivating for us to engage with, for instance, to gain physical fitness (Murrock and Gary, 2008, 2010; Hwang and Braun, 2015; Vahabi and Damba, 2015; Lukach et al., 2016; Serrano-Guzmán et al., 2016). Culture-based dances have been found to be effective in improving mobility, balance, and levels of physical fitness (Serrano-Guzmán et al., 2016), reducing blood pressure (Kaholokula et al., 2017), and diabetes (Murrock et al., 2009).

In addition to the cognitive health effects of dance practice, dancing also yields physical health effects. Dancing burns between 4 and 11 calories per minute, depending on the dance style (Williford et al., 1989), and increases heart rate up to around 140 BPM, making it an aerobic type of exercise for our body (Wigaeus and Kilbom, 1980; Blanksby and Reidy, 1988; Hogg et al., 2012; Nogueira et al., 2014; Cugusi et al., 2015). Dance practice has also been shown to have a positive effect on exercise ability and reduction of systolic (SBP) and diastolic (DBP) blood pressure, sleep and quality of life in people with hypertension and obesity (Mangeri et al., 2014; Conceição et al., 2016; Serrano-Guzman et al., 2016). It also boosts psychological and physical health of obese patients by increasing quality of life, body consciousness, mental representations linked to self-body image (Muller-Pinget et al., 2012; Allet et al., 2017), and cardiorespiratory capacity (Casilda-López et al., 2017).

Future research: cognitive and physical health measures vary considerably between different dance styles, something that might encourage differential assessments between different dance styles in the future (Lopez Castillo et al., 2015; Domene et al., 2016; see Box 4).

**Box 3.**

Dance and sociality.

**View from a dancer: sociality?**

Dance is an exemplary micro society in which nobody expects to be more right than the other because of his or her provenance, passport or birth place. It is not relevant where we come from, we speak a common language: dance, and that is sufficient. Dance and choreography make us equal humans while sharing a common experience. At the moment we come together to dance, it doesn’t matter who are we, where we come from, or how much money we have in our bank account. It is only important that we are fully present in our experience and open and communicative to share it with our companions in the moment. Therefore, dance breaks down social and economic barriers.

Luisa Sancho Escanero; Dresden-Frankfurt Dance Company, Germany

**View from a dancer: sociality?**

In today’s culture we are not accustomed to expressing ourselves through our body at any time we feel inclined. At the workplace we must maintain our physical composure to be considered “professional”; and in public places such as the market or the bank we must to the same lest we are seen as strange or even dangerous. It is only in specific social gatherings that we are permitted to let go of our composure- to an extent- and move in a way that would otherwise be considered inappropriate. These social events, such as parties and celebrations, provide us the opportunity to express joy and release tension by flailing our arms step in random or rhythmic patterns to the music, and throw our heads back and smile. These acts, done outside of the designated environments, would certainly get us in trouble, and perhaps get us labeled as mentally unstable. I cannot imagine what our lives would be like if we did not have these social outlets. Built-up tension and lack of emotional release would certainly lead to deep psychological and psycho-somatic issues. In our contemporary diverse communities, we meet people from different countries, and try to build relationships by learning about each other’s cultures and customs. Cultural subtleties that are difficult to understand through verbal explanations can be learned more effectively through movement and dance.

Shahrzad Khorsandi; Shahrzad Dance Company, San Francisco, United States

**Box 4.**

Dance and its many faces of technique and fitness. It happens that dancers become overly focussed on perfecting technique, on how they look, and they forget the important expressive exercises that would allow them to gain full potential from their practice (Karin, 2016; Karin et al., 2016). This finding merits further exploration in the future, to determine which aspects of dancing constitute health hazards for recreational dancers, and that obstruct the enjoyment of dancing.

**View from a dancer: What is the technique of a dance style?**

Any dance technique is our tool, our method, our skill, and our foundation. The technique of a dance style is the method we take, the more or less accurate approach. The codification of such a dance style: it is our grammar. But, we must not forget: it is a means not an end in itself. It is our path to freedom. We were taught that through the mastering of a dance technique (no matter which one in this case: classical, modern, contemporary…etc.) during our training years, we may get to enjoy more freedom and therefore, to enhance our capacity to focus intensively in the expression and/or interpretation of the dance. And this assumption is completely correct if we take classical ballet for this example. For more abstract contemporary ballet, the focus of the dancers is completely in the execution of the technique and through it, we achieve an abstract interpretation of the choreography. Historically, for instance in classical ballet, technique is considered the path to artistic freedom. The more technique I possess and/or I develop, equals to a bigger freedom in my execution. Therefore, I would be able to evolve higher artistically since I don’t have to worry about the execution of the choreography itself –which became a natural part of me, as if it was a new limb!

Luisa Sancho Escanero; Dresden-Frankfurt Dance Company, Germany

**View from a dancer: what happens when we practice a dance technique?**

Dance movements offer more variety in movement, a much bigger range of motion in the joints that do most sports or gym exercises. Increased range of motion in the joints leads to increased mobility and circulation and overall movement longevity. It may seem unnecessary for someone dancing for pure joy as a hobby, as opposed to preparing for a performance, to need technical training. However, technique does not have to be confined to a professional dance studio. It is simply the breaking down of any movement to its basic elements, such as geometric shapes, bodylines, foot patterns, and dynamic expression. This is especially true if the dance style is from a culture that is foreign to the dancer. It is intriguing, though not surprising, that common aesthetic elements exist in various art media within any particular culture within a given historical time period. The dances of a culture share aesthetics with other media of that culture, such as calligraphy, painting, textiles, sculpture, or architecture. These cultural aesthetics are woven into the fabric of the collective unconscious of both the artists and the viewers in that culture. Thus, the technique used in training a dancer in a particular dance form is informed by aesthetics that exist in the cultural context of the dance form. Technique and aesthetics are intertwined components of dance, because the goal of technique is to help the dancer gain mastery over her/his body in order to express him/herself with the language of aesthetics distinct to that culture and dance form. There is emotional gratification in both performing and watching movement performed with technical mastery and precision, resulting in an aesthetically pleasing dance.

Shahrzad Khorsandi; Shahrzad Dance Company, San Francisco, United States

### Connection and Connectedness

“Connection” is something that dancers refer to as a feeling that results from dancing together. It can refer to a connection with oneself as much as with the partner and/or group during the dance itself (Fitch, 2016; Christensen et al., 2017b), and/or to the music (Bernardi et al., 2017).

This is different from the social cohesion effect that we discussed in point section “Dance Styles” in that connectedness refers to a subjective impression that happens during the dance itself (even measurable via physiological synchronization such as breathing patterns among dancers Codrons et al., 2014), though some of the studies reported about in section “Dance Domains” specifically included questionnaire measures about feelings connectedness and subsequent trust in their assessments (Wiltermuth and Heath, 2009; Tarr et al., 2015). According to subjective reports from dancers, the stronger the connection during the dance, the more pleasing is the whole experience (Duberg et al., 2016; Bernardi et al., 2018). Another shade of this connectedness is the emotional link between audience and dancer that becomes established during some performances. Professional dancers may extend this feeling to the connectedness with the audience during a performance, a phenomenon that has even been related to a synchronization of physiological rhythms between dancer and audience (see e.g., Bachrach et al., 2015, for an exploration), however, this is not part of the current analysis.

Due to the strong emotional effects on the dancer of “feeling connected” during a dance (Himberg et al., 2018; Tarr et al., 2018), connectedness is an important phenomenon both for the dancer to focus on, but also, important for any researcher, seeking to research any effects produced by dancing, as the most important health effects of dancing are likely to occur only if genuine connectedness is perceived during the practice, i.e., it might not be enough to only “trail along.”

Dance can also connect people with a culture as it accounts for exclusive spiritual and cultural values and can be delivered within a familiar community setting that encourages a mind-body connection through traditional approaches which improves both physical and social functioning (Eyigor et al., 2009; Mavrovouniotis et al., 2010; Robinson et al., 2010; Zhang et al., 2013; Lukach et al., 2016; Kaholokula et al., 2017).

Future research: We are aware of no research that specifically explores this connection aspect of dance, though it seems of utmost importance for psychological wellbeing, if not also for physical health (see Box 5).

**Box 5.**

Dance and connectedness.

**View from a dancer: what does connectedness mean?**

When it comes to movement, I can think of three ways of connectedness: (1) connections between two or more bodies, (2) connections within one body, and (3) connection between a dancer and an outside entity. Whether a person is dancing in a “couple dance” such as Waltz or Tango, or dancing in a group, a connection is made between the bodies of the dancers through their common experience.

During dancing, one thinks, imagines, and feels (both emotion and kinetic sensation), while physically moving the body. Thus, there is a strong connection and interdependent relationship between the dancer’s mind and his/her body. This connectedness is dynamic as the sensations and imagery change during movement. Some dance forms imitate nature, such as the movement of cosmos or molecules, by moving in patterns that are circular and spiral. These organic movements satisfy a primal instinct by fostering a connection between the dancer (and also the viewer) and the movement found in nature. Also, in some dance forms a connection is made between the dancer and a spiritual entity, where the dancer is believed to become a channel of expression between the spirit and the audience. In all three types of connection, both the dancer and viewer often experience a sensation that is deep, rich, and cohesive, resulting in a “whole” experience that balances the mind-body interconnectedness.

Shahrzad Khorsandi; Shahrzad Dance Company, San Francisco, United States

**View from a dancer: how do dancers and audience connect?**

Humans have a tendency to search for connectedness. Take the example of a narrative classical ballet: if we dance together (24 women in a *corp de ballet* scene) we are feeling a living organism from inside, we are part of something bigger than each one of us. In this situation we are socializing (wanted or not), and connecting. We have to be aware not only of our movement, choreography, tasks, etc. We must be aware of our fellow dance colleagues, their steps, tasks, even their difficulties and/or possible mistakes. Spectators connect to us through empathy, empathy toward the ballet stories being dance on stage (in the case of classical narration) or through another kind of “aesthetic emotion”: the challenge of understanding and processing abstract choreographic information. The more of this abstract information being dance, the more attention it requires from the spectator. This is the catch, so to say.

Luisa Sancho Escanero; Dresden-Frankfurt Dance Company, Germany

### Flow and Mindfulness

Dancers often experience periods of flow and seek to increase the frequency of flow moments deliberately. For many dancers, their training is like a meditative practice.

Flow is a special state of consciousness that has been found related to many beneficial health effects (Csikszentmihalyi, 2008; Pfeifer et al., 2015). It is thus desirable to have flow moments in everyday life and dance has been proposed as an activity that produces flow (Vitebsky, 1995; Gore, 1997; Jeong, 2012). In particular, the feeling of “groove” during dancing has been found to be correlated with feelings of flow (Bernardi et al., 2018). Moreover, using imagery in dance practice increased the experience of flow (Jeong, 2012). However, it is still not entirely clear which aspect(s) of dance causes these flow effects.

Mindfulness meditation is difficult to describe for empirical research. However, in absence of a better definition, it can be described as a comprehensive wholesomeness training involves non-judgment, loving kindness and present-centered awareness (Marich and Howell, 2015). It requires concentration on aspects of the present by demanding a firm relation with the body and interoception (which is the perceptual process that provides us with the sense of inner body). A number of studies outside the dance realm revealed that mindfulness programs improved coping skills and self-regulation, decreased tendencies to take negative emotions of others (Beddoe and Murphy, 2004; Vago and David, 2012). Moreover, enhanced level of empathic concern was observed after mindfulness trainings (Lesh, 1970; Shapiro et al., 1998). Some dance forms are based on, or inspired by rituals and are by nature meditative and involving mindfulness. Could argue that any dance style that is practiced and performed can have meditative qualities for the dancer. The goal and definition of meditation is to contemplate or reflect upon a thought. In order for this to happen, the mind must be able to free itself of clutter and focus on the thought at hand. Similarly, a dancer’s mind must be free of clutter to be able to focus on embodying the movement exercises that are presented during class (see e.g., van Vugt, 2014; for such a first proposal regarding ballet dance). During dance practice, attention must be entirely focussed on the present moment and on one’s body from within, for instance, to be able to do a pirouette without falling over (Denardi and Correa, 2013), or to practice complex movement sequences, to balance. All these challenge the dancers’ attentional demands and helps keeping the attention in the here and now.

Mindfulness meditation has previously been proposed as an avenue to flow states. Some comparative studies assess several different dance forms in terms of how much the practitioners experience mindfulness and life satisfaction (Moyle, 2016). One study compared contemporary hobby dancers to a non-dance control group and found that the dance group reported high levels of mindfulness and life satisfaction (Muro and Artero, 2017), and a randomized controlled trial another found that Argentine tango classes were significantly related to decrease of depressive symptoms and increases in feelings of mindfulness (Dinzel, 1999; Pinniger et al., 2012).

Future research: Given the beneficial effects of mindfulness and flow experiences, and given first evidence that dance produces these states, future research might explore (1) which aspects of dancing give dancers flow and (2) whether there are differences between dance styles (very likely) (see Box 6).

**Box 6.**

Dance, flow and mindfulness.

**View from a dancer: How to get flow?**

Focussing on movement transitions (the moment when one movement stops and the next one begins) is significant to creating flow. To produce smooth transitions, the spatial pathways and planes of motions through which the body—or parts of the body—move at the moment of transition must be considered. In some art forms the characteristics of flow, ease, and effortlessness are essential, and help to create a mesmerizing or hypnotic effect on the audience, which is often the goal of the artist. In music, notes are sometimes suspended or played with a Legato quality to create smooth flow within the melody, and compositions are often cyclical, with progressions that produce melodic flow. A painter may use circular composition and techniques to dissolve one image into another, to produce smooth transformations of images. The viewer is then visually engaged by the circular motions of the composition and mesmerized by the smooth transformations of the images. In the same way, a dancer uses fluid and sweeping motions that surrender to gravity, and organic movement transitions, to create a flow that engages and mesmerizes the audience. This flow of movement can be very satisfying and even have physically and emotionally healing effects for both the dancer and the viewer.

Shahrzad Khorsandi; Shahrzad Dance Company, San Francisco, United States

**View from a dancer: How to get flow?**

I used to speak about “hyper reality” when referring to my moment performing on stage. Nothing else was happening in the Universe while I was on stage. It was only present existing: seconds last hours, and the most complicated choreographic tasks became slow motion in reality. There was a unique serenity and hyper-awareness while taking necessary artistic decisions. It is the creation of a reality in life. Those are the feelings on stage while performing. A creative way to get flow or “to stay in the flow” while executing very complex choreographies was/is to stay with our breath and try to keep it regular, no matter what difficulty we are going through physically at that very moment. To take our brain out of the complexity and make it focus in what is considered a mechanical function of the body. This was a thought (in my case, of course) which allowed me to achieve and deliver high complexity and at the same time, release my mind of a pressure that could paralyze me while trying to achieve the choreographic tasks. Besides, maybe due to this special flows state that dance gives us, dance/movement can make us change our current emotional state. E. g: being angry, depressed or upset at the beginning of a dance training. My emotional “mood” was completely transformed during the training. Toward the end of it, I could feel much more relaxed, calm and enjoying a positive “mood.”

Luisa Sancho Escanero; Dresden-Frankfurt Dance Company, Germany

### Aesthetic Emotions and the Imagination

Dancers use imagery and fantasy to improve their movements, and derive enjoyment from doing so. One type of enjoyment that dancers feel is aesthetic emotions like awe, being moved, tenderness etc. Such emotions can induce behavior change and a wish to grow and evolve.

The motor system of the human brain consists of different components that together give rise to the complex movements of a dance (sensorimotor cortices, sensory cortices, primary motor cortex, the premotor area, the supplementary motor area, the cerebellum and frontal association cortices; Carlson, 2004). All of these components are interconnected into feedback loops and it’s thanks to these loops that we’re able to move with such precision and grace, be it while walking down a narrow lane, or while dancing. The fact that the movement is “made” via feedback loops (and not, say, as a chain reaction), is a master piece of evolution, as it allows movements to be optimized online, i.e., while we move. We’re able to adjust our movements based on the ongoing sensory and motor feedback that keeps coming into the motor planner of our brain (Carlson, 2004; Karin et al., 2016). And this is also why the use of imagery to improve a dance movement is effective (Perica, 2010; Karin et al., 2016; Pavlik and Nordin-Bates, 2016).

It is not unusual to hear instructions in a dance class that tap into the brain’s capacity to act on imagined movements, sounds, metaphors or sensations. Instructions like “*move your arms like a swan*,” or “*straighten your spine as if I were pulling your hair upwards*”—make perfect sense to a dancer (recreational and professionals alike), while this might sound odd to the non-dancer.

Fortunately for those who have not yet tried to use their imagination to improve movements, this ability can be trained and improved with time (see also: Williams et al., 2013). Movement neuroscience has illustrated why such instructions are effective to optimize any movement. Imagined movements access the motor planner of our brain *directly*, without the slower and more error-prone feedback loop via the online sensory and motor input e.g., verbal and tactile feedback and corrections from a teacher). All movements that we have ever seen and can imagine, are stored to the greatest detail in our memory systems and are there to be used for movement optimization.

Janet Karin, OAM pioneered what she has coined Mental Training in dance teaching, and has differentiated and systematized the different types of imagery that are useful for dancers (Karin, 2016; Karin et al., 2016). Dancers can rely on internal or external motor imagery (Mahoney and Avener, 1977). Internal imagery means that the dancer imagines to be inside their body with all the sensations that it would entail to do a particular movement (Goldschmidt, 2002; Overby and Dunn, 2011). External imagery means that the dancer imagines being seen from the outside and feels how they would want the movement to unfold from the viewer’ (Golomer et al., 2008; Giron et al., 2012; Coker et al., 2015; Pavlik and Nordin-Bates, 2016).

As reviewed elsewhere (Karin, 2016; Karin et al., 2016; Christensen and Borhani, 2020), there are several types of imagery that assist all dancers in their practice. These include:

(i) *mental imagery* (imagine how the movement would look if it were completely correct). Dancers always rehearse choreographies mentally. They reproduce each step exactly with a mix of accuracy and idealism: they see in their minds the perfect execution of each choreographic step. For example, “my body is empty internally, I visualize only skeleton and joints exist in that **internal topography**.”
(ii) *visual imagery* (use an image of something in the physical world, e.g., “*move your arms in diagonal, as if you are shooting an arrow”*). Working with non-existing surfaces while dancing and/or transforming them mentally: “I open an imaginary door, I go around a corner that doesn’t exist to square a step, I flap my arms against surfaces that are not there to give the movement a specific density. Rest your head on the imaginary wall. I imagine the floor as shifting platforms that move against my limbs, thus not taking for granted the floor, and change my gravity while dancing.”
(iii) *kinesthetic imagery* (use physical sensations from the physical world: *“walk as if you’re smelling fresh bread”*). For example, “I imagine my lungs to be at my groins. My goal is to not jam myself against the floor with my movements because then, if my lungs are where my groins are I would suffocate. I imagine my eyeballs to be on my hands because in that way I could discover space with them in a different way while holding them in front of me. My hands with my eyeballs on them process the space and explore it in a brand new way.”
(iv) *metaphorical imagery* (use fantastic images, something that doesn’t quite make sense in the real world: “*move our arms like a swan’s wings*”). Any kind of animalist transformation: insects, animals in general, octopus with many limbs… For instance, “I have a million joints in my arms/legs and I must go through each one of them while moving my arms/legs (to over-articulate a movement). You are a 3,000 years old mummy coming back to life. Or I affect space with my metaphorical imagery, for example: I exit the stage, thinking that I am going to an endless, infinite space. This thought affects the quality of my leaving a scene.”
(v) *Musical imagery*. Dance is the only sport that systematically comes with music, and music is another way to imagery that can help with movement optimization. Music evokes imagery of many different forms by itself. Dancers can use imagery of auditory features (e.g., loudness or quiet), verbal imagery (words, dialog, interior monolog), nonverbal sounds imagery (e.g., musical melodies), and so on (Jakubowski, 2020). Imagined sounds, and other auditory events activate the same brain systems as if the sounds or events were really there (Hubbard, 2010). And many dancers can’t help it but imagine movements as soon as they hear music (Juslin and Vastfjall, 2008).
(vi) *Emotional imagination* is another category of imagery that helps the dancer optimize and enjoy their movements. For shy/introverted people, imagining that one is dancing completely alone (even if the room was full of people) can be reassuring and calming. When dancing, it can help to imagine the audience/those watching as if they are friends who give us a warm sense of approval, recognition and a feeling of being wrapped in that “home” sense. Our emotional states influence the way we move, walk, and do things. A sad person moves differently than an angry one, or than someone in love. With practice, using the vocabulary of a dance to intimate emotions into our movement, we automatically endow our movements the qualities of that emotion (Shafir et al., 2013; Shafir, 2016; Tsachor and Shafir, 2017). The motor commands composing these emotional movements are stored in our brain. The “Alexander Technique” (an acting technique) draws on this special capacity of our brain to make emotional movements. For instance, by recalling emotional episodes, the dancer can re-experience—through their imagination—what that episode felt like, and act it, or *dance* it *out* through their movements. This type of approach to emotions through dance affords important benefits in the therapeutic domain (Hanna, 1995; Jeong et al., 2005; Shafir et al., 2013, 2015; Koch et al., 2014; Shafir, 2016), because dancers can “try out” these emotions in the safe space of the dance studio (that is not the real world). Dance happens through the human body, as no other art form, and is therefore an instance of expressivity *par excellence*. Any emotion can be embodied by an individual in a dance. Just like any emotion that we feel may reflect itself in the way we walk, sit or do things at work and at home, dance movements are just prolongations of such everyday movements in that it allows the dancer to use an extended vocabulary of movements of a dance style, often aided by the musical accompaniment, to put emotional states into movement. This emotion-aspect of dance, that you can use dance as a way to intimate emotions, has been said to aid and develop emotion regulation abilities in practitioners (Shafir et al., 2013, 2015; Tsachor and Shafir, 2017).

**Box 7.**

Dance, the imagination and aesthetic emotions.

**View from a dancer: aesthetic demands?**

Should we free ourselves from aesthetic demands while dancing? I think this is a challenging question and a great debate to be taken in and developed. As I understand it, in any form of art, there are always aesthetic demands – even breaking all aesthetics demands it is in itself an aesthetic demand (which any avant-garde form of art tried to fulfill and/or achieved to fulfill). Dance is a human activity and, as such, evolves together with humans. We need to revisit our aesthetics demands in dance; we need to interrogate ourselves about our aesthetics expectations, how these demands are fulfilled and specially, with which quality toward ourselves these demands are fulfilled.

Luisa Sancho Escanero; Dresden-Frankfurt Dance Company, Germany

**View from a dancer: emotion and fantasy?**

Imagination plays a significant role in movement. We often imagine something before we can embody it. Imagery is used very often in dance, in order to express specific movement qualities or emotions. For example, to perform movement with an elongated quality (similar to the musical expression of Legato), a dancer may imagine as if she/he is moving in water, or honey, or “swimming in chocolate” to invoke imagery in my dance students that will help to convey this legato movement quality. There is a Persian dance movement that I refer to as *Ahiz (Aaheez*), which comes from an ancient Persian word referring to a warrior’s pulling a sword out of its scabbard, and lifting it with the intension of swinging it downward. While one arm lifts upward, the other arm stays low as an anchor, creating tension between the arms, and the spine lifts and elongates as the arm rises. Using the image of a sword being pulled out of its scabbard helps dancers create the necessary tension in the muscles to perform this movement with the correct quality.

Shahrzad Khorsandi; Shahrzad Dance Company, San Francisco, United States

The important point for researchers seeking to investigate the effects of recreational dancing is that the use of imagery is *very* commonplace, used every day in the practice, both to enhance the movements, but also, simply, because it’s fun. For the empirical scientist: What does it do to a person to continuously involve themselves in imagery and execute this imagery with their body? This is a very important component of any dance training; however, very little research explores this ability in terms of generalizability of skill to other domains of cognitive enhancement, for instance, together with the mnemotechnic tools that dancers develop to be able to remember large amounts of choreography (hour long performances).

As a side note: for the recreational dancer and teachers, this section should encourage them to use imagery of all kinds systematically and whenever possible to enhance movement and enjoyment alike.

Inseparable from any dance is the special aesthetics of each style that is learned with the technique. There is emotional gratification in both performing and watching aesthetically pleasing movement. Aesthetic emotions are normal, discrete, every-day emotions that we may feel in response to artworks, films, music, dances, architecture, nature scenes and many more situations and stimuli, whether we do these arts ourselves, or if we consume them (see the Multicomponent Model of Aesthetic Emotions Menninghaus et al., 2019, or one of the Vienna Models of Aesthetic Appreciation Leder et al., 2004; Pelowski and Akiba, 2011; Pelowski et al., 2016, 2017, and see also Chatterjee, 2003, 2011). When these emotions occur in the context of what the person experiences as an art that they derive an aesthetic experience from (and this may vary from individual to individual), an emotional distancing seems to occur, which enables us to derive enjoyment also from negative emotions, like, for instance, from a sad dance, and angry song, or a scary movie (Menninghaus et al., 2017). Aesthetic episodes activate the body’s physiology as normal emotional experiences do (e.g. quantifiable as changes in heart rate, pupillary responses, galvanic skin response, etc.), goose bumps/chills, shivers, tears (Pelowski and Akiba, 2011; Pelowski, 2015; Pelowski et al., 2016; Tinio and Gartus, 2018). The exploration of aesthetic emotions and their functions in dance are still scarce and is mostly focussed on liking and beauty ratings of observers (Calvo-Merino et al., 2008; Cross et al., 2011; Christensen and Calvo-Merino, 2013; Jola and Grosbras, 2013; Kirsch et al., 2015, 2016; Christensen et al., 2016b, 2018; Orgs et al., 2016; Reason et al., 2016), but very little empirical assessment is available about the dancers’ own aesthetic emotions as they dance (Bernardi et al., 2018). However, this is an area ripe for expansion, as aesthetic emotions are an important component of hobby dance (sport).

One mechanism that has been proposed to be at the core of dancers’ emotional advantages is interoception, because dancers and musicians have heightened interoceptive accuracy (Schirmer-Mokwa et al., 2015; Christensen et al., 2016a, 2017a). Interoception the sense that integrates bodily signals from within the body into a coherent whole, of “me,” as an autonomous agent in the world (selfhood) (Tsakiris, 2017). The experience-based interpretation of these signals (experiences as contingent to preceding events and other relevant contextual information, Seth, 2013; Seth and Critchley, 2013), has been proposed as a mechanism that affords emotional and homeostatic function (Craig, 2002, 2003, 2009; Critchley et al., 2004; Critchley, 2005, 2009). Studies have shown that interoceptive accuracy is positively related to components of a well-functioning person, such as emotional sensitivity (Dunn et al., 2010), empathy (Herbert et al., 2010), interpersonal sensitivity (Ferri et al., 2013), altruistic behavior (Weng et al., 2013), emotional resilience (Haase et al., 2016), and efficient decision-making under pressure (Werner et al., 2009, 2013; Wölk et al., 2014; Kandasamy et al., 2016).

However, in addition to such a physiological mechanism to boost emotional function, the regular experience of aesthetic emotions that have a strong motivational component (e.g., as a result of *being moved*, feeling *awe*, *tenderness*, etc.) might also make individuals more prone to behavior change and simply be more experienced in and used to *feeling*.

Future research: The effects of these types of imagery, of simulation and of physical and emotional enhancement through imagination is likely to induce changes at neurocognitive level, as is the frequent experience of aesthetic emotions. Such components of experience, however, are, to our knowledge, largely elusive in empirical research (see Box 7).

## Conclusion

Dance as a hobby affords many positive effects for the individual and their social group. However, more targeted research is needed that takes into account the important components that are part of any dance practice. Namely, effects of the music and rhythm on the individual, the sociality variables, the neurocognitive effect of the practice of technique and the special fitness effects derived from the exercise, the connectedness experiences, the flow and mindfulness aspects of the practice, and the imagery and aesthetic emotions that hobby dancers experience within and beyond practice.

Likewise, dance practitioners should embrace these various components more fully in their practice, since this allows a more holistic approach to the practice that will lead to increases in health and well-being, as shown by empirical research. Raising awareness and promoting discussion about these components within and across both scientific and dance domains is necessary for dance to become established as a fully recognized form of sport, while, at the same time, recognizing its artistic components fully, too (Vassallo et al., 2018).

Professional dancing, club dancing, religious and *erotic* dancing were specifically not considered within this paper, neither was Dance Movement Therapy (DMT).

Reductionistic views on dance as “for professionals only” or “for seduction only” have led to dance prohibitions of dance in different cultures throughout recorded history, e.g., in Japan, Germany, the United States, Iran, just to name a few. However, such perspectives are not aligned with modern scientific findings that suggest that many forms of dance afford health and wellbeing effects, over and above, any other sports and recreational activities. Furthermore, we have outlined that effects on the individual may depend on the dance style chosen (Shay, 1999; Verghese et al., 2003; Bremer, 2007; Merom et al., 2016; Christensen et al., 2017b). Awareness campaigns are strongly needed to educate the world’s societies on the benefits of recreational dance. It is up to future research to clarify the exact contributions of the different components that constitute recreational dances, and the specificities for each dance style to health and well-being effects.

## Author Contributions

JC, AG, SK, LE, and MV: conceptualization. JC, MV, and KB: literature search and manuscript preparation. SY, FF, and JC: figures. LE and SK: tables. All authors critical revisions and discussions of several rounds of manuscript.

## Conflict of Interest

LE was employed by the Dresden Frankfurt Dance Company. SK was employed by Shahrzad Dance Company (non-profit organization). FF and SY were employed by 3Fish corporate filmmaking. The remaining authors declare that the research was conducted in the absence of any commercial or financial relationships that could be construed as a potential conflict of interest.

## Footnotes

1 https://www.nyatango.com

2 https://www.tangoohlala.ch/tango-argentino
